# Epidemiological characteristics of injury among stand-up paddleboarding athletes

**DOI:** 10.1186/s40621-026-00667-4

**Published:** 2026-03-04

**Authors:** Hanyan Yan, Tingxv Zhang, Ziwen Mu, Haoxiang Wang, Zhiqiang Han, Huiru Ma, Kazuhiro Imai, Shaoshuai Shen, Hongtao Zeng, Xiao Zhou

**Affiliations:** 1 School of Physical Education, Huazhong University of Science and Technology, Luoyu Road 1037, Wuhan, 430074 China; 2 Department of Life Sciences, Graduate School of Arts and Sciences, The University of Tokyo, Komaba, Meguro-ku, Tokyo, 1538902 Japan; 3 School of Education and Welfare, Aichi Prefectural University, Aichi, Japan

**Keywords:** Competitive athletes, Stand-up paddleboarding, Paddle, Sports injury, Epidemiology

## Abstract

**Background:**

Stand-up paddleboarding has developed rapidly in recent years, but research on stand-up paddleboarding-related injuries is limited. Clarifying the characteristics of its injuries is needed to inform future prospective surveillance and prevention research.

**Purpose:**

This study aimed to investigate the distribution and incidence of self-reported stand-up paddleboarding-related injuries and to identify the paddling phases during which injuries were most frequently reported among stand-up paddleboarding athletes by age and gender.

**Methods:**

This study was a retrospective, self-reported injury survey conducted using a questionnaire among stand-up paddleboarding athletes aged 8–67 years who participated in municipal-level or above paddleboard events. An injury was defined as any physical complaint sustained during paddleboarding that caused: (a) missed training/competition, or (b) medical attention. Injury rates were calculated using Poisson distribution to determine the standardized incidence per 1000 training hours.

**Results:**

Among all the 330 athletes, 220 athletes (66.7%) reported 482 injuries. The shoulder was the most common injury site (20.1%, 97 cases), followed by the lower back (15.4%, 74 cases). Athletes experienced 5.39 sports injuries per 1000 training hours. Adult athletes had significantly higher injury rates than adolescent athletes (5.87 vs. 3.08 injuries per 1000 training hours). Adult athletes were more likely to sustain shoulder and lower back injuries than adolescent athletes. 50.6% (167 athletes) reported 355 injuries during paddling phases. The shoulder was the most common injury site during paddling phases (35.2%, 125 cases), followed by the lower back (23.4%, 83 cases). During the pull phase, 67.7% (113 athletes) of all the 167 athletes reported 155 injuries accounting for 43.7% of all phase-related injuries. The shoulder was the most common injury site in the pull phase (34.2%, 53 cases), while the lower back was the most common injury site in the exit phase (30.0%, 18 cases).

**Conclusion:**

The shoulder and lower back were the most common injury sites among competitive stand-up paddleboarding athletes, and injuries were most frequently reported during the pull phase of paddling. These descriptive findings may improve future injury prevention research and help athletes and coaches enhance safety awareness.

## Background

Stand-up paddleboarding originated in Hawaii in the 1950s as a combination of surfing and paddling [1], attracting a growing number of participants through recreational and competitive activities, with its popularity experiencing exponential growth [2]. The paddling cycle comprises four phases: catch, pull, exit, and recovery [3]. To maintain paddling smoothly, athletes require simultaneously activating and coordinating muscles in the upper limbs, torso, thigh, and lower limbs [4]. During paddling, the paddler grips the shaft with the hand on the paddling side while holding the T-grip with the opposite hand. By extending the shoulders and torso, and coordinating angular changes in the shoulder, elbow, hip, and knee joints on both sides of the body, the paddler drives the paddle backward to complete the stroke, thereby propelling the board forward [5–7]. Paddlers with different experience levels demonstrate distinct technical characteristics, with inexperienced athletes proven to rely more heavily on shoulder and elbow movement to execute paddling strokes [8]. Repetitive strain disorder induced or exacerbated by force application [9].

Epidemiological studies showed that over 50.0% of injuries among stand-up paddleboarding athletes were overuse, with nearly 60.0% of injuries occurring in the upper limbs or thorax region [10]. The injury rate in stand-up paddleboarding was 3.63 per 1,000 h of participation. Among 240 paddleboarding participants, 95 reported at least one injury. The most frequently injured areas were the shoulder joint and upper arm (32.9%), followed by the lower back (14.3%), and the elbow joint and forearm (11.8%) [11]. In one study involving 72 injured participants, 50.0% of stand-up paddleboarding-related injuries required hospital treatment, and 20.0% resulted in absence from work [12].

However, epidemiological research on stand-up paddleboarding related injuries remains limited, particularly in Chinese athlete populations which have expanded rapidly in recent years, and phase-stratified descriptions across the stroke cycle are scarce in the existing literature. Sports injuries can result in musculoskeletal deformities and asymmetry between bilateral limbs in athletes, particularly young athletes, preventing continued sports participation [13]. Therefore, preventing, reducing, and controlling stand-up paddleboarding-related injuries is a critical prerequisite for injury-free participation and maintaining a healthy, active lifestyle [14].

To enhance injury prevention, prolong athletic careers, and improve competitive performance among stand-up paddleboarding athletes, this study aimed to: (1) investigate the characteristics and incidence of the injuries among competitive stand-up paddleboarding athletes by age and gender, (2) identify the injury characteristics during paddling phases.

## Materials and methods

### Methods

This study was a retrospective, self-reported injury survey conducted using a structured questionnaire. Athletes completed paper questionnaires offline from March 2024 to April 2025 in Hubei, Chongqing, Hunan, Zhejiang, and other event regions during competitions, including the 2024 Asian Paddleboard Championships, the 2024 National Paddleboard Championships, the 2024 Hubei Provincial Paddleboard Championships, the 2024 Changsha City Paddleboard Competition, the 2025 Zhejiang Provincial Paddleboard Rescue Championships. Concurrently, electronic questionnaires were distributed online via website links sent to athletes by contacting event organizers. Minors (< 18 years) were required to complete the questionnaire under guardian supervision. All participants received information regarding the study objectives and completion procedures. Each participant signed an informed consent form prior to participation.

Paper questionnaires were administered to athletes with at least one hour of availability before, during, and after competitions. Throughout the survey process, research hypotheses were not disclosed, and participants were explicitly informed of the purpose of the survey. Participants were instructed to provide responses neutrally based on their actual experiences. A total of 386 stand-up paddleboarding athletes (aged 8 to 67 years) participated in the survey. After screening for eligibility and data completeness, 330 questionnaires were retained for analysis (valid response rate: 85.5%). Accordingly, 330 competitive Chinese stand-up paddleboarding athletes were included based on the following criteria: (1) participation in at least one municipal-level or higher paddleboard competition, (2) minimum one year of training experience, (3) training frequency of at least once per week, and (4) completed questionnaires without abnormal response.

The questionnaire collected variables used in the data analysis: (1) participation characteristics and stand-up paddleboarding training exposure (e.g., years of experience, training frequency, and training duration) for estimating total exposure time; (2) stand-up paddleboarding-related injuries occurring within the 48 weeks preceding questionnaire completion, with injury location recorded using a standardized body image with predefined anatomical sites; and (3) stroke-phase reporting, in which participants indicated the paddling phase (catch, pull, exit, and recovery) during which the reported injury occurred or was most commonly provoked. This study was reviewed and approved by the Institutional Ethics Board of Tongji Medical College, Huazhong University of Science and Technology, China (Notification Number [2023] IEC (S172). The research protocol was conducted in accordance with the principles of the Declaration of Helsinki.

### Injury definition

Based on the International Olympic Committee (IOC) injury surveillance system and adapted to this study’s cohort, stand-up paddleboarding athletes meeting any of the following criteria during participation were defined as injured: (1) resulting in absence from training sessions or competitions; (2) requiring medical intervention during training/competition [15]. Meeting one or more of them was counted as one time, and multiple injuries in the same part were recorded repeatedly.

### Statistical analysis

The Shapiro-Wilk test was used to examine the normality of basic parameters. Age, height, weight, body mass index (BMI), years of training experience, annual training hours, cumulative training hours, total training sessions, number of trainings per week, and training session duration did not follow a normal distribution.

Data were analyzed using the Mann-Whitney U test, with participants divided into two age groups: adults (≥ 18 years) and adolescents (< 18 years), and two gender groups: male and female. The injury incidence rate per 1,000 h of paddleboarding was calculated using Poisson distribution:

Injury incidence per 1,000 training hours = (Total number of injuries / Total training time per year) × 1000;

Total training time per year = Training hours per session × Number of trainings per week × 48;

Total number of training sessions per year = Number of trainings per week × 48.

*Chi-square* tests were performed to compare injury incidence of body regions by age and gender. Odds ratios (OR) with 95% confidence intervals (95% CI) were calculated. Further, logistic regression models were performed to examine associations between paddleboarding-related injuries and variables. Three models were specified: Model 1 included gender and age; Model 2 included gender, age, height, and BMI; and Model 3 included gender, age, height, BMI, years of training, number of trainings per week, training time per session, and total training time. Multicollinearity was assessed using variance inflation factors (all VIF < 5). For all statistical analyses, *p-value* < 0.05 was considered statistically significant,

## Results

Among all the 330 competitive Chinese stand-up paddleboarding athletes, there were 276 adults (≥ 18 years) and 54 adolescents (< 18 years), with 211 males and 119 females.

Table 1 shows that adult athletes showed significantly greater values than adolescent athletes in age (*p* < 0.001), height (*p* < 0.001), weight (*p* < 0.001), BMI (*p* < 0.001), years of training (*p* < 0.001), number of trainings per week (*p* < 0.01), number of trainings per year (*p* < 0.05), and total number of training sessions (*p* < 0.001). Adolescent athletes reported longer training time per session than adult athletes (*p* < 0.001). No significant differences were observed between adult and adolescent athletes in training time per year and total training time.

Male athletes showed significantly greater values than females in height, weight, BMI, training time per year, number of trainings per week, number of trainings per year, total number of training sessions, and total training time (all *p* < 0.001). No significant differences were found in years of training and training time per session between them.

**Table 1:** Basic information on stand-up paddleboarding athletes broken down by gender and age

Variable	Total (*N* = 330)	Adult (*N* = 276)	Adolescent (*N* = 54)	Male (*N* = 211)	Female (*N* = 119)
Age (years)	34.9 ± 15.9	39.1 ± 13.8^***^	13.3 ± 1.9	34.8 ± 15.4	35.2 ± 16.8
Height (cm)	168.4 ± 9.6	170.0 ± 8.2^***^	160.6 ± 12.0	172.5 ± 8.2^†††^	161.1 ± 7.1
Weight (kg)	67.0 ± 17.2	69.6 ± 15.9^***^	53.8 ± 17.3	72.0 ± 17.1^†††^	58.3 ± 13.5
BMI (kg/m²)	23.5 ± 5.2	24.0 ± 4.8^***^	20.8 ± 6.2	24.1 ± 5.2^†††^	22.4 ± 5.6
Years of training (years)	3.2 ± 2.0	3.4 ± 2.1^***^	1.9 ± 1.1	3.3 ± 2.1	2.8 ± 1.7
Training time per year (hours)	270.8 ± 332.9	268.4 ± 350.4	282.7 ± 225.0	312.0 ± 391.4^†††^	197.7 ± 167.1
Number of trainings per week (times)	3.0 ± 2.3	3.1 ± 2.4^**^	2.5 ± 2.1	3.3 ± 2.6^†††^	2.5 ± 1.7
Training time per session (hours)	1.8 ± 1.0	1.7 ± 1.0^***^	2.4 ± 0.8	1.9 ± 1.1	1.6 ± 0.7
Number of trainings per year (times)	143.3 ± 112.9	147.9 ± 114.4^*^	120 ± 102.9	157.9 ± 124.6^†††^	117.6 ± 82.9
Total number of training sessions (times)	468.1 ± 559.3	510.8 ± 554.4^***^	249.3 ± 537.6	533.9 ± 624.5^†††^	351.3 ± 395.8
Total training time (hours)	854.5 ± 1405.6	911.5 ± 1455.0	562.7 ± 1084.2	1018.0 ± 1655.0^†††^	564.5 ± 708.1

Values are presented as mean ± standard deviation. ^***^p < 0.001, ^**^p < 0.01, and ^*^p < 0.05 between Adults vs. adolescent athletes; ^†††^*p* < 0.001 between male vs. female athletes

In total, 66.7% (*n* = 220) of all the participants, including 190 adults and 30 adolescents reported 482 paddleboarding-related injuries (adults: 435 injuries; adolescents: 47 injuries). 66.8% (*n* = 141) of 211 male athletes reported at least one injury, while 66.4% (*n* = 79) of 119 female athletes reported at least one injury.

As for injured anatomical site, the shoulder ranked first (*n* = 97, 20.1%), followed by lower back (*n* = 74, 15.4%), and knee (*n* = 44, 9.1%). Among adult athletes, shoulder (*n* = 92, 21.1%) was the most frequent injured site, followed by lower back (*n* = 71, 16.3%) and knee (*n* = 40, 9.2%), while among adolescent athletes, ankle (*n* = 6, 12.8%) was the most frequent injured site, followed by shoulder and scapula (*n* = 5 each, 10.6%), and knee (*n* = 4, 8.5%). Among male athletes as well as among female athletes, the shoulder (male: *n* = 67, 20.3%; female: *n* = 30, 19.7%) ranked first, followed by lower back (male: *n* = 50, 15.2%; female: *n* = 24, 15.8%) and knee (male: *n* = 25, 7.6%; female: *n* = 19, 12.5%) (Table 2).

**Table 2:** Distribution of stand-up paddleboarding–related injuries by gender and age

Site	Total *N*(%)	Adult *N*(%)	Adolescent *N*(%)	Male *N*(%)	Female *N*(%)
Face	3(0.6)	2(0.5)	1(2.1)	2(0.6)	1(0.7)
Chest	9(1.9)	9(2.1)	0(0.0)	7(2.1)	2(1.3)
Abdomen	6(1.2)	5(1.1)	1(2.1)	5(1.5)	1(0.7)
Shoulder	97(20.1)	92(21.1)	5(10.6)	67(20.3)	30(19.7)
Elbow	22(4.6)	20(4.6)	2(4.3)	17(5.2)	5(3.3)
Wrist	21(4.4)	21(4.8)	0(0.0)	12(3.6)	9(5.9)
Palm/Fingers	19(3.9)	17(3.9)	2(4.3)	15(4.5)	4(2.6)
Thigh joints	7(1.5)	7(1.6)	0(0.0)	6(1.8)	1(0.7)
Anterior thigh	11(2.3)	8(1.8)	3(6.4)	7(2.1)	4(2.6)
Knee	44(9.1)	40(9.2)	4(8.5)	25(7.6)	19(12.5)
Anterior lower leg	4(0.8)	4(0.9)	0(0.0)	4(1.2)	0(0.0)
Ankle	17(3.5)	11(2.5)	6(12.8)	8(2.4)	9(5.9)
Toes	10(2.1)	7(1.6)	3(6.4)	5(1.5)	5(3.3)
Head	2(0.4)	1(0.2)	1(2.1)	1(0.3)	1(0.7)
Neck	10(2.1)	9(2.1)	1(2.1)	8(2.4)	2(1.3)
Scapula	38(7.9)	33(7.6)	5(10.6)	24(7.3)	14(9.2)
Back	31(6.4)	29(6.7)	2(4.3)	22(6.7)	9(5.9)
Lower back	74(15.4)	71(16.3)	3(6.4)	50(15.2)	24(15.8)
Hip	9(1.9)	8(1.8)	1(2.1)	6(1.8)	3(2.0)
Upper arm	14(2.9)	12(2.8)	2(4.3)	10(3.0)	4(2.6)
Forearm	7(1.5)	7(1.6)	0(0.0)	6(1.8)	1(0.7)
Posterior thigh	6(1.2)	5(1.1)	1(2.1)	5(1.5)	1(0.7)
Posterior lower leg	6(1.2)	5(1.1)	1(2.1)	6(1.8)	0(0.0)
Achilles tendon	6(1.2)	3(0.7)	3(6.4)	4(1.2)	2(1.3)
Foot	9(1.9)	9(2.1)	0(0.0)	8(2.4)	1(0.7)
Total	482(100.0)	435(100.0)	47(100.0)	330(100.0)	152(100.0)

Data are presented as number of cases (percentage %), with injuries at each site counted by frequency of occurrence. Multiple injury sites could be reported by the same athlete

**Fig. 1:**
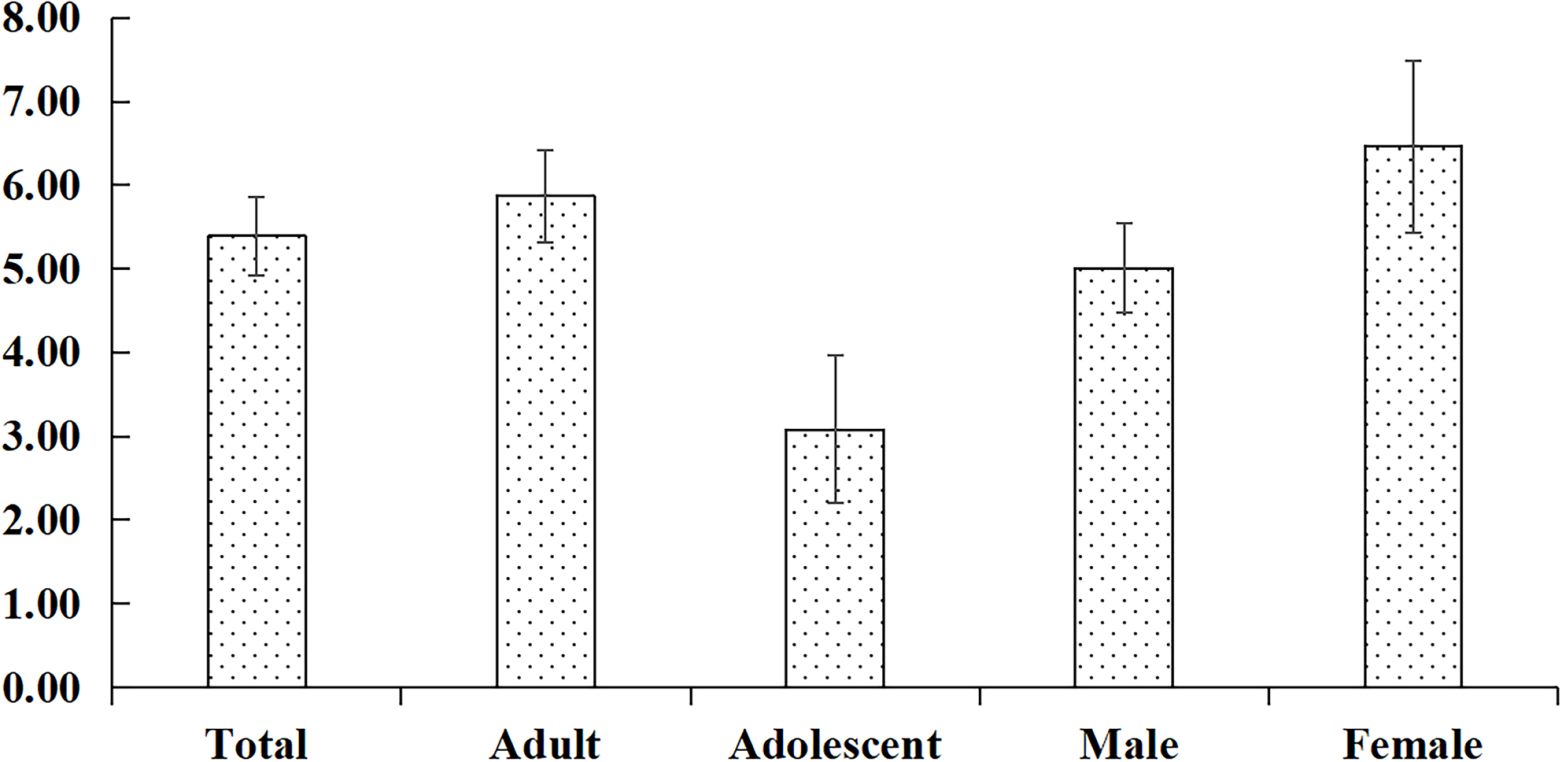
Injury incidence per 1,000 training hours among stand-up paddleboarding athletes. Error bars indicate 95% confidence intervals

The injury incidence rate per 1000 training hours among stand-up paddleboarding athletes was 5.39 (95% CI: 4.92–5.87). By age, adult athletes showed an injury rate of 5.87 (95%CI: 5.32–6.42) per 1000 training hours which was significantly greater than adolescent athletes with an injury rate of 3.08 (95%CI: 2.20–3.96) per 1000 training hours. By gender, male athletes showed an injury rate of 5.01 (95%CI: 4.47–5.55) per 1000 training hours, and female athletes showed a rate of 6.46 (95%CI: 5.43–7.49) per 1000 training hours (Fig. 1).

167 athletes (50.6%) reported 355 injuries during the paddling phase, with shoulders (35.2%), lower back (23.4%), and elbows (9.3%) being the most common areas. During catch phase, 89 athletes reported injuries; during pull phase, 113 athletes reported injuries; during exit phase, 44 athletes reported injuries; and during recovery phase, 31 athletes reported injuries.

Catch phase: 101 injuries (28.5%), top three sites: shoulder (38 cases, 37.6%), lower back (22 cases, 21.8%), elbow (12 cases, 11.9%); pull phase: 155 injuries (43.7%), top three sites: shoulder (53 cases, 34.2%), lower back (36 cases, 23.2%), elbow (16 cases, 10.3%); exit phase: 60 injuries (16.9%), top three sites: lower back (18 cases, 30.0%), shoulder (16 cases, 26.7%), palm/fingers (4 cases, 6.7%); recovery phase: 39 injuries (11.0%), top three sites: shoulder (18 cases, 46.2%), lower back (7 cases, 17.9%), elbow (4 cases, 10.3%) (Table 3).

**Table 3:** Injury distribution by paddleboarding stroke phase

Site	Catch *N*(%)	Pull *N*(%)	Exit *N*(%)	Recovery *N*(%)
Abdomen	0(0.0)	0(0.0)	0(0.0)	1(2.6)
Shoulder	38(37.6)	53(34.2)	16(26.7)	18(46.2)
Elbow	12(11.9)	16(10.3)	1(1.7)	4(10.3)
Wrist	4(4.0)	6(3.9)	3(5.0)	0(0.0)
Palm/Fingers	1(1.0)	5(3.2)	4(6.7)	0(0.0)
Thigh joints	0(0.0)	0(0.0)	0(0.0)	1(2.6)
Anterior thigh	1(1.0)	0(0.0)	2(3.3)	0(0.0)
Knee	7(6.9)	6(3.9)	3(5.0)	1(2.6)
Anterior lower leg	0(0.0)	2(1.3)	2(3.3)	1(2.6)
Ankle	1(1.0)	0(0.0)	1(1.7)	1(2.6)
Toes	0(0.0)	0(0.0)	1(1.7)	0(0.0)
Head	0(0.0)	1(0.6)	1(1.7)	1(2.6)
Scapula	2(2.0)	8(5.2)	3(5.0)	2(5.1)
Back	6(5.9)	9(5.8)	2(3.3)	2(5.1)
Lower back	22(21.8)	36(23.2)	18(30.0)	7(17.9)
Hip	0(0.0)	0(0.0)	1(1.7)	0(0.0)
Upper arm	4(4.0)	9(5.8)	1(1.7)	0(0.0)
Forearm	1(1.0)	4(2.6)	0(0.0)	0(0.0)
Posterior lower leg	1(1.0)	0(0.0)	1(1.7)	0(0.0)
Achilles tendon	1(1.0)	0(0.0)	0(0.0)	0(0.0)
Total	101(100.0)	155(100.0)	60(100.0)	39(100.0)

Data are presented as number of cases (percentage %). Injuries at different body sites are ranked by frequency of occurrence. To improve readability, categories with zero counts across all four stroke phases are not displayed in the table

Compared with adolescent athletes, adult athletes were 5.89 times more likely to sustain lower back injuries (OR = 5.89, 95%CI: 1.78–19.46; *χ²* = 10.56, *p* = 0.001), 4.9 times likely to sustain shoulder injury (OR = 4.9, 95%CI: 1.89–12.72; *χ²* = 12.61, *p* < 0.001). Adults were 0.33 times likely to sustain ankle injury than adolescents (OR = 0.332, 95%CI: 0.12–0.94; χ² = 4.693, *p* = 0.030); lower back injury risk in adults was 5.89 times than that of adolescents, and Achilles tendon injury risk in adults was 0.19 times than that of adolescents (OR = 0.187, 95%CI: 0.04–0.952; *χ²* = 5.052, *p* = 0.025). *Chi-square* tests comparing body injury sites between 211 male athletes and 119 female athletes showed no significant differences in injury sites (*p* > 0.05).

Multicollinearity diagnostics indicated no collinearity in Model 1 (two variables). In Model 2, multicollinearity was detected; weight showed the highest VIF (70.369) and was therefore removed, after which all remaining variables had VIF values < 5. In Model 3, multicollinearity was detected; Total training sessions showed the highest VIF (23.778) and was removed, but collinearity persisted. Training time per year then showed the highest VIF (9.882) and was removed; all remaining variables subsequently had VIF values < 5. Accordingly, Model 1 included gender and age; Model 2 included gender, age, height, and BMI; and Model 3 included gender, age, height, BMI, years of training, number of trainings per week, training time per session, and total training time. Multivariable logistic regression analysis indicated a modest association between number of trainings per week and injury occurrence. (OR = 1.40, 95% CI 1.07–1.84; *p* = 0.016) (Table 4).

**Table 4:** Logistic regression analysis of factors associated with injury occurrence

Variable	Model1 OR (95%CI)	*p*	Model2 OR (95%CI)	*p*	Model3 OR (95%CI)	*p*
Gender						
Male	1.00	-	1.00	-	1.00	-
Female	0.98(0.61–1.58)	0.936	0.99(0.55–1.78)	0.982	1.17(0.63–2.17)	0.627
Age (years)	1.00(0.99–1.01)	0.977	1.00(0.99–1.02)	0.906	1.00(0.99–1.02)	0.692
Height (cm)	-	-	1.00(0.97–1.03)	0.846	1.01(0.98–1.04)	0.649
BMI (kg/m²)	-	-	0.99(0.94–1.04)	0.624	0.99(0.95–1.04)	0.649
Years of training (years)	-	-	-	-	0.99(0.80–1.23)	0.938
Number of trainings per week (times)	-	-	-	-	1.40(1.07–1.84)	**0.016**
Training time per session (hours)	-	-	-	-	0.87(0.68–1.12)	0.277
Total training time (hours)	-	-	-	-	1.00(1.00-1.11)	0.447

Bold p-values indicate significant. BMI is body weight and height calculation; OR: Odds Ratio; 95%CI: 95% Confidence Interval; Multicollinearity diagnostics indicated acceptable collinearity (all VIF < 5)

## Discussion

To the best of our knowledge, this study was the first epidemiological investigation among competitive Chinese stand-up paddleboarding athletes, revealing that over half of the participants sustained at least one paddleboarding-related injury, with the shoulder and lower back being the most frequently injured sites. Adult athletes demonstrated significantly higher injury rates than adolescents, along with increased risks for shoulder and lower back injuries. The adolescent group was smaller than the adult group (54 vs. 276 participants), which limits the precision of age-group estimates. Age-group differences may also reflect cumulative training exposure (e.g., years of participation and accumulated training load) in addition to chronological age; accordingly, we interpreted these as observed associations. During the pull phase, athletes exhibited the highest injury incidence, with the shoulder being the most vulnerable site, while the lower back was the most common injured site during the exit phase. A significant association was observed between the number of trainings per week and the occurrence of stand-up paddleboarding-related injuries.

An epidemiological study of paddleboarding-related injuries among 240 stand-up paddleboarding athletes found that the overall injury rate was 70.0% [10]. Regarding other paddling sports, an epidemiological study on kayaking using offline and online questionnaires found that among 392 athletes with a median age of 34 years, over half of them sustained at least one injury during paddleboarding [16]. Another epidemiological study on kayaking employed a prospective follow-up approach to investigate injuries among 63 kayaking athletes, revealing an injury rate exceeding 50.0% [17]. Findings from previous studies consistently indicated that over half of athletes experienced at least one injury, which aligns with the results of our studies.

Regarding injury incidence per 1000 training hours, an epidemiological study on paddleboarding reported that participants had an injury rate of 3.63 injuries per 1000 h (95% CI: 3.04–4.16) [10]. Another epidemiological study on paddleboarding reported that among 86 stand-up paddleboarding athletes who participated in competitions, the injury rate was 3.21 injuries per 1000 h [4]. In this study, we found that 330 athletes had an injury rate of 5.39 injuries per 1000 h (95% CI: 4.92–5.87), which is significantly higher than the previous studies, which may be related to differences between the participants in this study (mean age 34.9 years and 100% competition participation) and the participants in the previous study (mean age 45 years and 19% competition participation; mean age 43.7 years and 67.2% competition participation) [10, 17].

Meanwhile, previous studies reported that athletes sustaining sports injuries were older than healthy athletes [18]. In a study on age distribution characteristics of paddleboarding injuries, investigation of injury rates among stand-up paddleboarding athletes aged 8–82 years revealed that across three age groups: < 40 years (15.2% injury incidence), 41–50 years (17.1%), and > 50 years (17.9%), older age groups showed progressively higher injury rates [12]. Another study also showed higher injury rates among athletes > 46 years [10]. In this study, the injury rate of adult athletes was significantly higher than that of adolescents, which is consistent with the previous studies.

Regarding training volume, indicating the relationship between training volume and injuries in paddleboard athletes with sports injuries versus healthy paddleboard athletes, showed that significant differences existed in weekly training volume, weekly training days, training duration per session, and other aspects [19]. Existing research has confirmed that training hours was a key factor causing sports injuries [20]. Athletes with practice time less than 10 h have lower injuries than those with practice time of 10 h or longer. Longer practice time leads to greater accumulated fatigue [21]. The greater the total training volume, the higher the injury risk; the more the frequency, the higher the injury rate [22]. This may help contextualize the observed association between the number of trainings per week and stand-up paddleboarding-related injury occurrence.

Regarding anatomical sites, one study on paddleboarding reported that shoulder/upper arm (32.9%) and lower back injuries were high-incidence sites in paddleboard athletes [10]. Shoulder and lower back injuries commonly appear in water sports similar to stand-up paddleboarding. One study reported that among 1214 water sports athletes, paddle-related injuries frequently occurred in shoulder (31.0%) and lower back (23.5%) [15]. Another study reported 392 elite dragon boat athletes, with the most common injury sites being shoulder (16.33%), hand (12.29%), and lower back (11.24%) [13]. This study found that shoulder and lower back were the most common injury sites in stand-up paddleboarding athletes, which is consistent with previous findings.

Regarding age and injury sites, previous research on stand-up paddleboarding on athletic conditions found that older stand-up paddleboarding athletes were more prone to injuries [23], especially shoulder and lower back injuries [10]. One study further investigated whether age differences in stand-up paddleboarding athletes affected shoulder injury risk, finding that older paddleboard participants had a higher shoulder injury risk than younger paddleboard participants [13]. Regarding the relationship between age and lower back injuries, no paddleboarding-related research was found. A study on the similar sport of surfing found that among 1348 surfers, older and professional surfers faced higher injury risks compared to younger and recreational surfers [24]. Other sports, such as baseball, discovered that the prevalence of lower back pain increases with increasing age [25]. This study also found that shoulder and lower back injury incidence rates in adults were higher than in adolescents, consistent with existing research.

Shoulder overuse during paddling has been discussed in relation to rotator cuff pathology in kayaking/rowing populations, which may provide a plausible overuse mechanism to contextualize the shoulder injuries reported in stand-up paddleboarding. Situations may originate from the movement characteristics of paddleboarding. Athletes need to activate trunk, upper limbs, hip, knee, and ankle muscles to achieve more efficient paddling motions. Force transfers from lower limbs to upper limbs, causing the upper limbs to sustain greater load [10, 17, 26]. Biomechanical research on kayaking paddling found that when kayak athletes moved the blade backward in the pull phase, maximum pull was generated through trunk rotation and shoulder joint extension movements, accompanied by active electromyography activity in the upper trapezius muscle [27]. In stand-up paddleboarding biomechanical studies, both experienced and inexperienced stand-up paddleboarding athletes exhibited certain ranges of joint motion changes in the shoulder and hip joints to complete forward propulsion through alternating left/right strokes during paddling [27, 28]. Kayak athletes also perform alternating left/right strokes to complete paddling motions [29]. Shoulder overuse during paddling is one of the causes of rotator cuff injuries in marathon kayakers [30]. During paddleboarding, the pull phase accounts for 50.0% of the entire paddling cycle, being the longest among the four phases [2].

Trunk rotation is a critical biomechanical consideration in paddle sports. Firstly, during blade entry (catch phase), trunk rotation engages the abdominal and lumbar muscles to initiate the stroke. Secondly, trunk rotation maximizes blade propulsion force and stroke length, thereby optimizing paddling efficiency [13]. Trunk rotation prolongs the duration for which the paddle blade maintains an effective, perpendicular position relative to the water surface. Mann and Kearney (1980) identified that the core paddling action involves a coordinated sequence: the push phase of the thrust segment combined with the pull phase of the recovery segment, integrated with trunk rotation. This synergy rapidly positions the blade vertically within the pull zone and maximizes its dwell time in this position, enhancing stroke efficiency [31]. Research using 3D motion capture to assess the range of motion (ROM) in stand-up paddleboarding athletes demonstrated great trunk rotation angles during paddling, consistently observed in both experienced and novice participants [27]. Excessive trunk rotation has been described as a potential risk factor for lower back symptoms in related paddle sports [32], which may offer theoretical context for the lower back injuries reported in our studies. In this study, phase-attributed injuries were most frequently reported during the pull phase. Shoulder injuries were most commonly reported during the pull phase, whereas lower back injuries were most commonly reported during the exit phase.

No studies on the correlation have been found between the exit phase of stand-up paddleboarding athletes and injuries. The electromyography of stand-up paddleboarding athletes showed that the back muscles were active during the exit phase, which lasts for 13.0% [33]. Epidemiological explanations for lower back injuries have been explained in related water rowing, with excessive lower back flexion and extension proposed as important contributing mechanisms [32]. which could support our findings that stand-up paddleboarding athletes have a high incidence of lower back injury.

### Limitations

This study was a retrospective, self-reported injury survey among stand-up paddleboarding athletes (aged 8–67 years) and has several limitations. First, retrospective self-reporting may introduce recall bias, particularly for minor injuries. Stroke-phase attribution was also based on retrospective self-report and may be imprecise. Second, injury incidence was calculated using estimated training exposure derived from self-reported typical training frequency and session duration, and a 48-week multiplier was used to approximate annual training exposure, which may affect the precision and cross-study comparability of incidence estimates. Third, the sample may be influenced by self-selection and by potential underrepresentation of athletes with more severe injuries who were unable to participate in training or competitions at the time of survey completion. Fourth, the adolescent subgroup was substantially smaller than the adult subgroup (54 vs. 276 participants), which may limit the precision and stability of age-stratified comparisons. Fifth, injuries were not clinically verified, which may introduce misclassification and limit diagnostic specificity. Finally, because logistic regression was specified at the athlete level (injured vs. not injured), the analysis did not model repeated injury events within individuals; future studies could apply injury-level approaches when repeated events are analyzed. Prospective studies with objectively recorded exposure and clinical verification are warranted to improve measurement precision and comparability across cohorts.

To address these constraints, future investigations should implement prospective designs incorporating wearable biometric monitors (e.g., inertial measurement units, surface electromyography) synchronized with digital training logs to objectively quantify exposure variables. A tripartite injury verification protocol is recommended: (1) athlete symptom reporting, (2) clinical examination by sports medicine practitioners, and (3) confirmatory imaging where clinically indicated. Targeted expansion of youth cohorts through stratified sampling by developmental stage (pre-/post-pubertal) is essential, complemented by structured coach interviews to identify technique-mediated risk factors. These methodological refinements will enable sophisticated time-to-event analyses of training load-injury relationships and evidence-based prevention program development.

## Conclusion

This study provides descriptive epidemiological data on stand-up paddleboarding–related injuries, including injury distribution by anatomical site and stroke-phase attribution. Adults reported a higher injury incidence than adolescents, and weekly training times were associated with injury occurrence. These findings can help stand-up paddleboarding athletes pay attention to injuries to body sites while paddling and further guide the development of targeted prevention strategies.

## Data Availability

The datasets used and/or analyzed during the current study are available from the corresponding author on reasonable request.

## References

[1] Ruess C, Kristen KH, Eckelt M, Mally F, Litzenberger S, Sabo A. Stand up paddle surfing—an aerobic workout and balance training. Procedia Eng. 2013;60:62–6.

[2] Hammer S. Catch the wave of stand up paddling. Providence J. 2011;7:3.

[3] Michael JS, Smith R, Rooney KB. Determinants of kayak paddling performance. Sports Biomech. 2009;8(2):167–79. 19705767 10.1080/14763140902745019

[4] Tsai FH, Wu WL, Chen YJ, Liang JM, Hou YY. Electromyography analysis of muscle activation during stand-up paddleboarding: a comparison of paddling in kneeling and standing positions. Appl Sci. 2020;10(7):2356.

[5] Schram B, Hing W, Climstein M. Profiling the sport of stand-up paddleboarding. J Sports Sci. 2016;34(10):937–44. 10.1080/02640414.2015.1079331. 26289320 10.1080/02640414.2015.1079331

[6] Schram B. Stand up paddle boarding: an analysis of a new sport and recreational activity [doctoral thesis]. Gold Coast (Australia): Bond University; 2016.

[7] Yan H, Mu Z, Zhang T, Imai K, Ma Y, Zhou X. Assessment of kinematic characteristics for stand-up paddleboard motor skills. In: Proceedings of the International Conference on Physical Education and Sport Science (ICPESS). Macau; 2024.

[8] Schram B, Furness J, Kemp-Smith K, Sharp J, Cristini M, Harvie D, et al. A biomechanical analysis of the stand-up paddleboarding stroke: a comparative study. PeerJ. 2019;7:e8006. 31695968 10.7717/peerj.8006 PMC6827442

[9] Lessenger JE, editor. Agricultural medicine: a practical guide. New York (NY): Springer Science+Business Media; 2006.

[10] Castañeda-Babarro A, Calleja-González J, Viribay A, Fernández-Lázaro D, León-Guereño P, Mielgo-Ayuso J. Relationship between training factors and injuries in stand-up paddleboarding athletes. Int J Environ Res Public Health. 2021;18(3):880. 33498553 10.3390/ijerph18030880 PMC7908629

[11] Furness J, Olorunnife O, Schram B, Climstein M, Hing W. Epidemiology of injuries in stand-up paddleboarding. Orthop J Sports Med. 2017;5(6):2325967117710759. 28638840 10.1177/2325967117710759 PMC5472235

[12] Balke M, Fischer M, Kegler T, Höher J, Balke M. Injuries and use of safety equipment in stand-up paddle boarding. Orthop J Sports Med. 2021;9(8):23259671211022681. 10.1177/23259671211022681. 34471645 10.1177/23259671211022681 PMC8404644

[13] Maffulli N, Longo UG, Gougoulias N, Caine D, Denaro V. Sport injuries: a review of outcomes. Br Med Bull. 2011;97(1):47–80. 20710023 10.1093/bmb/ldq026

[14] Verhagen EALM, van Stralen MM, van Mechelen W. Behaviour, the key factor for sports injury prevention. Sports Med. 2010;40:899–906. 20942507 10.2165/11536890-000000000-00000

[15] Junge A, Engebretsen L, Alonso JM, Renström P, Mountjoy M, Aubry M, et al. Injury surveillance in multi-sport events: the International Olympic Committee approach. Br J Sports Med. 2008;42(6):413–21. 18390916 10.1136/bjsm.2008.046631

[16] Fiore DC, Houston JD. Injuries in whitewater kayaking. Br J Sports Med. 2001;35(4):235–41. 11477016 10.1136/bjsm.35.4.235 PMC1724359

[17] Toohey LA, Drew MK, Bullock N, Caling B, Fortington LV, Finch CF, et al. Epidemiology of elite sprint kayak injuries: a 3-year prospective study. J Sci Med Sport. 2019;22(10):1108–13. 31239203 10.1016/j.jsams.2019.06.002

[18] Jayanthi NA, LaBella CR, Fischer D, Pasulka J, Dugas LR. Sports-specialized intensive training and the risk of injury in young athletes: a clinical case-control study. Am J Sports Med. 2015;43(4):794–801. 25646361 10.1177/0363546514567298

[19] Kirkhorn SR, Earle-Richardson G. Repetitive motion injuries. In: Lessenger JE, editor. Agricultural medicine: a practical guide. New York (NY): Springer Science+Business Media; 2006. pp. 324–38.

[20] Drew MK, Finch CF. The relationship between training load and injury, illness and soreness: a systematic and literature review. Sports Med. 2016;46(6):861–83. 26822969 10.1007/s40279-015-0459-8

[21] Prieto-González P, Martínez-Castillo JL, Fernández-Galván LM, Casado A, Soporki S, Sánchez-Infante J. Epidemiology of sports-related injuries and associated risk factors in adolescent athletes: an injury surveillance study. Int J Environ Res Public Health. 2021;18(9):4857. 34063226 10.3390/ijerph18094857 PMC8125505

[22] Jones BH, Cowan DN, Knapik JJ. Exercise, training and injuries. Sports Med. 1994;18(3):202–14. 7809556 10.2165/00007256-199418030-00005

[23] Nathanson A, Haynes P, Galanis D. Surfing injuries. Am J Emerg Med. 2002;20(3):155–60. 11992332 10.1053/ajem.2002.32650

[24] Bacanac LR, Radovic M, Veskovic A. Frequency of sports injuries depending on gender, age, sports experience, nature of sports and training process. Serbian J Sports Sci. 2007;1(4):122–8.

[25] Kato K, Otoshi K, Tominaga R, Kaga T, Igari T, Sato R, et al. Age-related differences in the limited range of motion of the lower extremity and their relation to lower back pain in young baseball players: a cross-sectional study. Sports Med Open. 2023;9(1):26. 37138150 10.1186/s40798-023-00572-w PMC10156885

[26] Ho SR, Smith R, O’Meara D. Biomechanical analysis of dragon boat paddling: a comparison of elite and sub-elite paddlers. J Sports Sci. 2009;27(1):37–47. 19031333 10.1080/02640410802491350

[27] Pelham TW, Robinson MG, Holt LE. Injuries in sprint canoeists and kayakers: etiology, mechanisms of injury, treatment options, and practical applications. Strength Cond J. 2020;42(3):22–9.

[28] Castañeda-Babarro A, Viribay A, León-Guereño P, et al. Anthropometric profile, body composition, and somatotype in stand-up paddleboarding international athletes: a cross-sectional study. Nutr Hosp. 2020;37(5):958–63. 32960636 10.20960/nh.03021

[29] Begon M, Colloud F, Sardain P. Lower limb contribution in kayak performance: modelling, simulation and analysis. Multibody Syst Dyn. 2010;23(4):387–400.

[30] Hagemann G, Rijke AM, Mars M. Shoulder pathoanatomy in marathon kayakers. Br J Sports Med. 2004;38(4):413–7. 15273173 10.1136/bjsm.2002.003699 PMC1724871

[31] Mann RV, Kearney JT. A biomechanical analysis of the Olympic-style flatwater kayak stroke. Med Sci Sports Exerc. 1980;12(3):183–8. 7402054

[32] Rumball JS, Lebrun CM, Di Ciacca SR, Orlando K. Rowing injuries. Sports Med. 2005;35(6):537–55. 10.2165/00007256-200535060-00005. 15974636 10.2165/00007256-200535060-00005

[33] Ruess C, Kristen KH, Eckelt M, Mally F, Litzenberger S, Sabo A. Activity of trunk and leg muscles during stand-up paddle surfing. Procedia Eng. 2013;60:57–61.

